# MR-guided transcranial focused ultrasound safely enhances interstitial dispersion of large polymeric nanoparticles in the living brain

**DOI:** 10.1371/journal.pone.0192240

**Published:** 2018-02-07

**Authors:** David S. Hersh, Pavlos Anastasiadis, Ali Mohammadabadi, Ben A. Nguyen, Sijia Guo, Jeffrey A. Winkles, Anthony J. Kim, Rao Gullapalli, Asaf Keller, Victor Frenkel, Graeme F. Woodworth

**Affiliations:** 1 Department of Neurosurgery, University of Maryland School of Medicine, Baltimore, Maryland, United States of America; 2 Department of Diagnostic Radiology and Nuclear Medicine, University of Maryland School of Medicine, Baltimore, Maryland, United States of America; 3 Marlene and Stewart Greenebaum Comprehensive Cancer Center, University of Maryland School of Medicine, Baltimore, Maryland, United States of America; 4 Department of Surgery, University of Maryland School of Medicine, Baltimore, Maryland, United States of America; 5 Center for Vascular and Inflammatory Diseases, University of Maryland School of Medicine, Baltimore, Maryland, United States of America; 6 Department of Pharmacology, University of Maryland School of Medicine, Baltimore, Maryland, United States of America; 7 Department of Anatomy and Neurobiology, University of Maryland School of Medicine, Baltimore, Maryland, United States of America; Universidad de Castilla-La Mancha, SPAIN

## Abstract

Generating spatially controlled, non-destructive changes in the interstitial spaces of the brain has a host of potential clinical applications, including enhancing the delivery of therapeutics, modulating biological features within the tissue microenvironment, altering fluid and pressure dynamics, and increasing the clearance of toxins, such as plaques found in Alzheimer’s disease. Recently we demonstrated that ultrasound can non-destructively enlarge the interstitial spaces of the brain *ex vivo*. The goal of the current study was to determine whether these effects could be reproduced in the *living* brain using non-invasive, transcranial MRI-guided focused ultrasound (MRgFUS). The left striatum of healthy rats was treated using MRgFUS. Computer simulations facilitated treatment planning, and targeting was validated using MRI acoustic radiation force impulse imaging. Following MRgFUS treatments, Evans blue dye or nanoparticle probes were infused to assess changes in the interstitial space. In MRgFUS-treated animals, enhanced dispersion was observed compared to controls for 70 nm (12.8 ± 0.9 mm^3^ vs. 10.6 ± 1.0 mm^3^, p = 0.01), 200 nm (10.9 ± 1.4 mm^3^ vs. 7.4 ± 0.7 mm^3^, p = 0.01) and 700 nm (7.5 ± 0.4 mm^3^ vs. 5.4 ± 1.2 mm^3^, p = 0.02) nanoparticles, indicating enlargement of the interstitial spaces. No evidence of significant histological or electrophysiological injury was identified. These findings suggest that transcranial ultrasound can safely and effectively modulate the brain interstitium and increase the dispersion of large therapeutic entities such as particulate drug carriers or modified viruses. This has the potential to expand the therapeutic uses of MRgFUS.

## Introduction

Approximately 15–20% of the total volume of the brain is comprised of an anisotropic, narrow, and tortuous interstitial/extracellular space (ECS) [1]. Movement of fluids and substances within the brain is determined in part by diffusion within the ECS as well as bulk flow through perivascular spaces. As a result, alterations of the physical characteristics and microstructures of the brain ECS, including diameter (d_ECS_) and tortuosity (λ), may affect a variety of physiological and pathophysiological processes. The diffusion of morphogens during embryogenesis [2], the movement of neurotransmitters during neuronal signaling [3–5], and the dispersion of therapeutic agents within the brain parenchyma may be limited by the narrow diameter and high tortuosity of the ECS [1]. Structural alterations of the brain ECS and perivascular spaces may also impact the function of the glial-lymphatic system (GLS), a recently described pathway for the clearance of interstitial solutes and waste products from within the brain [6–9]. Dysfunction of the GLS has been implicated in a number of neuro-degenerative conditions, including aging [10], Alzheimer’s disease [7, 11–13], and traumatic brain injury [14].

Several groups have investigated methods to modify the brain ECS, including enzymatic degradation of the extracellular matrix and dilation of the ECS via hyperosmotic solutions, in an effort to improve the distribution of locally delivered therapeutic agents [15–18]. Recently, however, transcranial focused ultrasound (FUS) has emerged as a technology with the potential to non-invasively modify the structure and properties of the brain ECS. FUS can alter biological tissues via thermal and mechanical mechanisms. In sound-absorbing media, continuous exposures produce heat, resulting in coagulative necrosis and thermal ablation of soft tissues [19]. Having recently gained FDA approval for the treatment of Essential Tremor, non-invasive transcranial MR-guided FUS (MRgFUS) mediated ablation of deep brain targets is currently being investigated for the treatment of other neurological disorders, including Parkinson’s disease and neuropathic pain [20]. In other applications, pulsed FUS exposures with short duty cycles will generate lower temporal-averaged rates of energy deposition, producing minimal temperature elevations (≤ 5°C) [21]. In the absence of large temperature elevations, pulsed FUS allows for the mechanical effects of ultrasound to predominate, including cavitation, radiation forces, and acoustic streaming [22–24]. Transcranial FUS is being widely investigated for its ability to transiently disrupt the blood-brain barrier (BBB) in the presence of intravascular microbubbles (MBs). The MBs undergo stable oscillations in the presence of an acoustic field, localizing the effects of FUS to the cellular components that contribute to the BBB [25].

In addition to these blood vessel-directed effects with FUS-MB combinations, transcranial FUS alone may also generate mechanical effects directly on the brain parenchyma. Radiation force-induced displacements and the resulting strain within the tissue may stretch or disrupt the tissue at its weakest point—likely the intercellular protein complexes that tether adjacent cells [23]. This can increase the space between cells and within the extracellular matrix. We observed similar effects in muscle [23], tumor xenografts [26], and more recently, in fresh brain slices [27]. In the latter work, our group examined the movement of nanoparticle probes to study changes in the spaces between brain cells and adjacent to blood vessels after treatment with non-destructive ultrasound. Increases in the perivascular and interstitial spaces were observed, resulting in increased mobility of nanoparticle probes as large as ~500 nm within the brain tissue. Whether similar FUS exposures could be applied to the living brain through the intact skull, and if similar findings would be detected following treatments *in vivo*, remained key questions to uncovering the potential value of this non-thermal ultrasound regimen.

In this study, we examined the effects of transcranial FUS *in vivo* using an MRgFUS system with an array transducer, similar to clinical MRgFUS systems [28]. The MR-guided device enables the transcranial deposition of pulsed ultrasound energy within the rodent brain. We studied the distribution of a small molecule, Evans blue dye (EBD), as well as nanoparticle probes of different diameters in the living brain following MRgFUS treatment. Furthermore, we incorporated computer simulations to assist in the treatment planning, evaluated magnetic resonance acoustic radiation force impulse (MR-ARFI) imaging for target validation, and examined the electrophysiological and histopathological changes in response to the treatments.

## Materials and methods

### Simulation of the focal zone

The acoustic properties of the skull, brain and water are shown in Table 1, and were applied to simulations of the acoustic field [29]. A non-linear acoustic model was employed to solve the Westervelt equation [30] as shown below:

$$\rho_{0}\nabla ∙(\frac{1}{\rho_{0}}\nabla p)-\frac{1}{{c_{0}}^{2}}\frac{\partial^{2}p}{\partial t^{2}}+\frac{\delta }{{c_{0}}^{4}}\frac{\partial^{3}p}{\partial t^{3}}+\frac{\beta }{\rho_{0}{c_{0}}^{4}}\frac{\partial^{2}p}{\partial t^{2}}\;=\;0,$$ (1)

where *p* is the acoustic pressure; *c*_*0*_ is the speed of sound; ρ_0_ is the density; β = 0.1 is the acoustic nonlinearity coefficient; δ is the sound diffusivity. The sound diffusivity δ is obtained through the relationship $\alpha \;=\;\delta \omega^{2}/2c_{0}^{3}$, where α is the acoustic attenuation coefficient and ω is the angular frequency of ultrasound. A 1.5 MHz sinusoidal waveform was applied to the ultrasound transducer.

**Table 1:** Compressional acoustic properties of skull, brain tissue and water (free field).

Speed of sound [m/s]		Absorption [dB (MHz cm)^-1^]		Density [kg/m^3^]	
*C*_bone_	2500	*α*_bone_	0.67	*ρ*_bone_	1500
*C*_brain_	1560	*α*_brain_	0.34	*ρ*_brain_	1030
*C*_water_	1480	*α*_water_	2.5 × 10^−5^	*ρ*_water_	1000

### Transcranial MRgFUS exposures

All *in vivo* animal experiments were carried out using 4-week-old Sprague Dawley rats (Envigo RMS, Indianapolis, IN) weighing 100–120 g, in accordance with NIH guidelines for animal welfare as well as protocols approved by the University of Maryland School of Medicine Institutional Animal Care and Use Committee (protocol #0816021). The rats were socially housed in plastic bins with wire tops and corn-cob bedding (24.3 cm × 19 cm × 18 cm) and received food and water ad libitum, with a room temperature of 23°C and a 12 hour light/dark cycle. Animals were monitored daily for signs of distress (e.g. failure to groom, decreased social interaction), neurotoxicity, weight loss, pain or discomfort.

Non-invasive FUS exposures were applied using a commercial MRgFUS system (Image Guided Therapy, Pessac, France) designed to safely provide targeted transcranial FUS exposures to the rodent brain. MRI guidance was performed using a 7.4T animal scanner (Bruker, Hamburg, Germany). During the procedure, animals were anesthetized with 1.5%-2.0% vaporized isoflurane and 100% oxygen (flow rate = 1 L hr^-1^) delivered through a nosecone. Physiological monitoring was performed with an SAII MRI compatible unit (Small Animal Instruments Inc., Stony Brook, NY).

The MRgFUS system has an RF coil built into the animal holder. The 8-element, annular array 1.5 MHz FUS transducer (Imasonic, Voray-sur-l’Ognon, France) was positioned over the shaved head of the animal. The transducer was coupled to the head via an acoustically transparent membrane that maintained a small volume of degassed water between the face of the transducer and the head. A thin layer of ultrasound coupling gel was applied between the membrane and the head.

Once the anesthetized animal was secured in the holder and the transducer was placed over its head, the assembly was inserted in the bore of the MRI scanner. The animal was kept warm with a heated water jacket, and its respiration rate was monitored remotely, with the level of anesthesia varied accordingly. Baseline T2-weighted images were obtained to ensure the head was in the geometric center of the scanner and that proper coupling had been achieved, using the following parameters: repetition time = 3000 ms, echo time = 36 ms, slice thickness = 1 mm, number of slices = 30. The images were transferred from the scanner to the GUI of the MRgFUS system, and the position of the transducer was reconstructed to overlay on axial and coronal images for targeting.

The region to be treated was selected using the remote positioning capabilities of the transducer in all 3 axes. The focal zone of the transducer was remotely positioned at the selected brain region using two servo motors that moved it in the *x*-*y* plane with an accuracy of 1 mm via the system’s GUI. The GUI was similarly used to position the focal zone in the *z* axis by varying the applied power to a combination of 8 annular transducer elements.

MR-ARFI exposures were applied at acoustic pressures of 1.95, 2.25, and 2.4 MPa each in 5 separate animals. 3 ms pulses were provided at a duty cycle of 3%, for a total of 1200 pulses. Post-processing of the MR-ARFI planes generated a color map indicating the treated region. The brain tissue displacement by ultrasound-induced radiation forces was calculated for each of the three exposure amplitudes [31].

For treatments preceding the infusion of EBD and nanoparticle probes, as well as for histological and electrophysiological comparisons, FUS exposures were carried out in pulsed mode using 10 ms pulses provided at a 10% duty cycle and pulse repetition frequency of 10 Hz for 2 min, with a pressure amplitude of 2.3 MPa at the focal zone (I_SATP_ 35 W/cm^2^). Rastering through 15 points (3 × 5) in the *x*-y plane generated a treatment volume encompassing the ipsilateral striatum. Raster points were 1.5 mm apart in each dimension.

### Nanoparticle preparation and characterization

Non-adhesive nanoparticles were prepared as described previously [32–34]. Briefly, blue-dyed COOH-modified polystyrene (PS) nanoparticles (Bangs Laboratories Inc., Fishers, IN) 70 nm, 200 nm, and 700 nm in diameter were modified with methoxy-PEG5k-amine (Creative PEGWorks, Winston Salem, NC) by a carboxyl-amine reaction. The PEGylated nanoparticles were prepared from stock solutions and were suspended in deionized water at concentrations of 25 mg/ml, 12.5 mg/ml, and 12.5 mg/ml for 70 nm, 200 nm, and 700 nm nanoparticles, respectively. The physicochemical properties of the nanoparticles were characterized with a Zetasizer NanoZS instrument (Malvern Instruments, Southborough, MA). The hydrodynamic diameter, ζ-potential, and polydispersity index (PDI) of the nanoparticles were measured via dynamic light scattering and laser Doppler anemometry (Table 2).

**Table 2:** Physiochemical properties of non-adhesive polystyrene nanoparticles.

Estimated particle diameter (nm)	Measured particle diameter (nm)^a^	ζ-potential (mV)^b^	Polydispersity index
70	71.5 ± 0.2	-6.7 ± 0.4	0.18
200	224.0 ± 1.7	-3.4 ± 0.2	0.03
700	682.4 ± 4.2	-3.2 ± 0.3	0.04

^a^ Diameter (number mean) measured via dynamic light scattering. Data represents the average of 3 independent measurements ± SEM, with n ≥ 100 particles per measurement.

^b^ Measured at 25°C in 15× diluted PBS (10mM NaCl; pH 7.4). Data represents the average of 3 independent measurements ± SEM, with n ≥ 100 particles per measurement.

### Local delivery of dye or nanoparticles

*In vivo* infusions (EBD or nanoparticles) were carried out by modifying a protocol described previously [17]. MRgFUS-treated animals and age-matched controls were anesthetized with 1.5%-2.0% vaporized isoflurane and 100% oxygen, while a heating pad maintained body temperature. The animal was secured in a stereotactic frame, after which an incision was made along the dorsal midline of the cranium. A 1-mm burr hole was then drilled +0 mm anterior and 2.5 mm lateral to the bregma, and a Hamilton syringe was continuously inserted to a depth of 4.5 mm at a rate of 1 mm/s. These coordinates were used to target the infusion to the striatum, corresponding to the center of the region that underwent sonication via MRgFUS. The syringe was left in place for 2 minutes prior to initiation of the infusion. The plunger of the syringe was attached to a Nanojet stereotaxic syringe pump (Chemyx Inc., Stafford, TX), and 2.5 μL of EBD or nanoparticles were infused at a rate of 0.1 μL/min. Following completion of the infusion, the syringe was left in place for an additional 2 minutes, and was then slowly removed.

The animals underwent euthanasia 2 hours following completion of the infusions. The animals underwent cardiac perfusion with normal saline and 4% paraformaldehyde (PFA) while being administered inhalant anesthesia, followed immediately by craniocervical dislocation and decapitation. The brains were removed and stored in Optimal Cutting Temperature (OCT) embedding compound (Sakura Tissue Tek Inc., Torrance, CA) at -80°C. The brains were then sectioned in the coronal plane into 10 μm slices on a Leica CM3050 S cryostat (Leica Biosystems Inc., Buffalo Grove, IL). A digital camera was used to obtain high-resolution images, beginning with the first appearance of EBD or nanoparticles, and proceeding with every sixth frozen brain slice until the EBD or dyed particles were no longer visible.

### Image processing

Image processing and analysis was performed via modification of a protocol described previously [35]. The images were imported into MATLAB (Mathworks, Natick, MA). Each coronal image was cropped around the brain with a rim of white OCT compound, to generate a 530 x 380 pixel image. Due to differences in ambient light intensity during the capture of high-resolution images from multiple experiments, each image underwent an automated normalization process. The Red-Green-Blue (RGB) 24-bit depth color intensity values of reference points within the brain tissue, distant from the injection site, were extracted. The RGB values of the pure white OCT compound were then divided by the average RGB values of the reference points, in order to obtain a correction factor for each color channel. The correction factors were applied to the images. The RGB images were then converted to grayscale. Otsu thresholding was used to identify the optimal threshold for each image in a fully automated fashion, as previously described [36]. Briefly, the maximum between-class variance method was used to split each image into a “foreground” and “background” and identify the threshold value which minimized the sum of foreground and background pixel spreads. A binary image was then generated and the total area of distribution was then calculated. The volume of distribution was calculated by multiplying the area of distribution in each image by the thickness of the slice (10 μm), multiplying the product by 6 (as images were obtained of every sixth slice), and summing the results for all of the images for each individual brain.

### Histological analysis

A separate cohort of MRgFUS-treated animals (n = 4) and age-matched controls (n = 4) underwent cardiac perfusion with 4% PFA, after which the brains were harvested and preserved in 4% PFA for 24 hours. The brains were subsequently transferred to a 70% ethanol solution followed by paraffin embedding, sectioning, and H&E staining. The stained slices were assessed for evidence of histological damage by a neuropathologist who was blinded to the treatment status.

### Electrophysiological experiments

A separate cohort of MRgFUS-treated animals (n = 5) and age-matched controls (n = 5) were anesthetized with ketamine/xylazine, their brains removed, and horizontal slices (300 μm thick) containing the striatum were prepared, following the method described by Ting et al. [37] For recordings, slices were placed in an interface chamber and continually perfused (2 ml/min) with artificial cerebrospinal fluid (ACSF) containing, in mM: NaCl (119), KCl (2.5), NaH_2_PO_4_ (1.2), NaHCO_3_ (2.4), glucose (12.5), MgSO_4_•7H_2_O (2), CaCl_2_•_2_H_2_O (2).

Whole-cell patch clamp recordings were obtained through pipettes containing, in mM, potassium gluconate (120), potassium chloride (10), HEPES (10), magnesium chloride (1), ATP-Mg (2.5), EGTA (0.5), GTP-Tris (0.2). Impedance of patch electrodes was 4 to 6 MΩ. Series resistance (<40 MΩ, compensated at least 60%) was monitored throughout the recording, and recordings were discarded if series resistance changed by more than 20%. All recordings were obtained at room temperature.

### Statistical analysis

All values are presented as mean ± standard deviation (SD). Student’s unpaired *t* tests were used for pairwise comparisons. For multiple comparisons, two-way ANOVA was performed. All tests were performed at the 0.05 level of significance. All statistical analyses were performed with Prism (GraphPad Software, Inc., La Jolla, CA).

## Results

### Simulation of the acoustic intensity field

**Fig 1:**
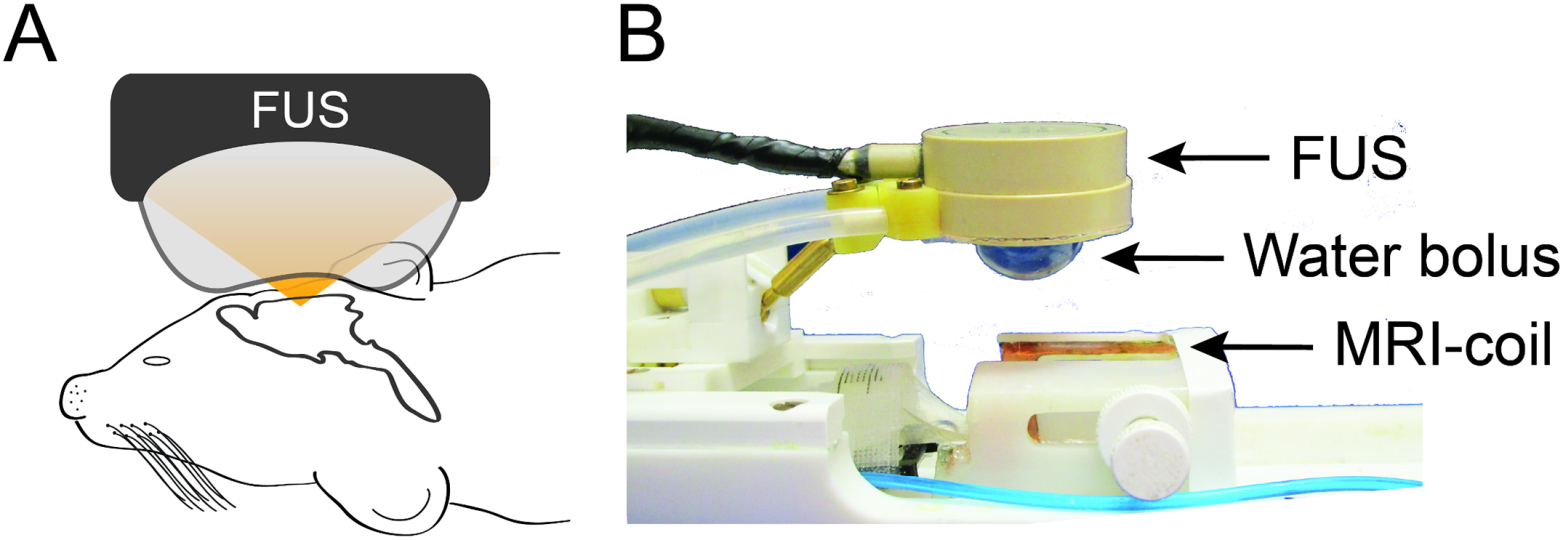
Schematic representation of the MRgFUS system. (A) The animal’s head was positioned between the MRI coil and the FUS water bolus. (B) The RF coil is built into the animal holder of the system. An 8-element, 1.5 MHz FUS transducer was positioned over the head of the animal. The transducer was coupled to the head via an inflated membrane containing degassed water.

**Fig 2:**
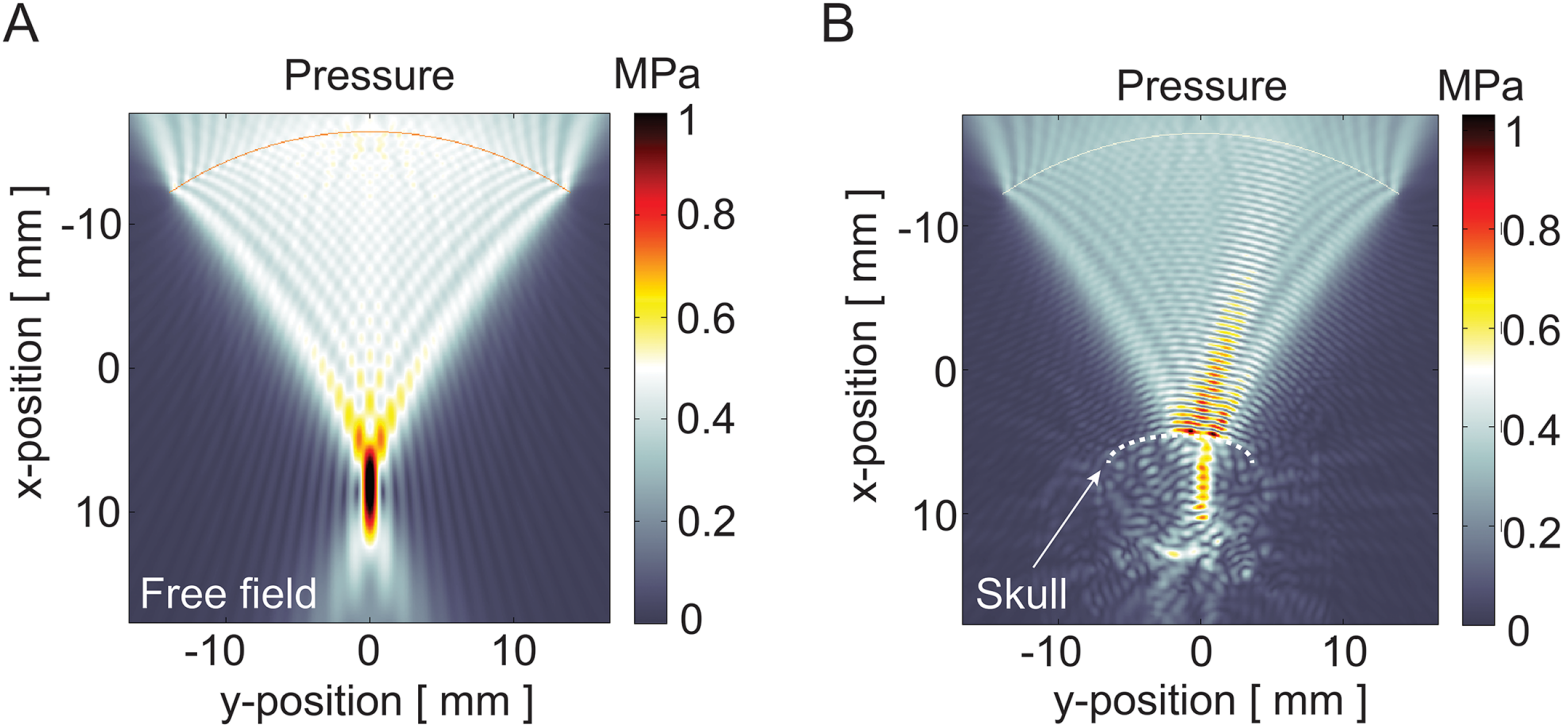
Acoustic pressure field simulations. Acoustic pressure fields generated by a 1.5 MHz FUS 8-element annular array transducer in (A) free field and (B) in the setting of the *in vivo* transcranial treatments. The dashed line outlines the contour of the skull.

All FUS treatments in this study were carried out using a commercial, MR-guided system designed to provide transcranial FUS exposures (Fig 1). In order to predict the acoustic pressure fields generated by this system, simulations were performed that accounted for the transducer dimensions and frequency, the skull dimensions, and the density, ultrasound velocity, and absorption coefficients of the treated and intervening tissues (Table 1). Normalized acoustic intensity fields were simulated for the 1.5 MHz multi-array FUS transducer in free field and in the setting of the *in vivo* treatments within the rodent brain, where the skull was present in the pre-focal region. This was achieved by solving the wave propagation equation, where acoustic pressure maps were generated illustrating the lateral and vertical peak acoustic pressure profile of the acoustic beam in the focal plane (Fig 2). The simulations revealed that although the skull absorbs and reflects a component of the acoustic energy, the transmitted energy remains focused along an elongated elliptical zone between the skull and the target. A focal zone with a radial diameter of 1 mm and an axial diameter of 10 mm was found, the latter being similar in dimension to the cranial-caudal diameter of the brain among the animals used in this study. These acoustic intensity field simulations were used to develop a rastering scheme that achieved complete sonication of the striatum using the least number of discrete raster points and therefore, the shortest treatment time.

### Magnetic resonance acoustic radiation force impulse imaging

**Fig 3:**
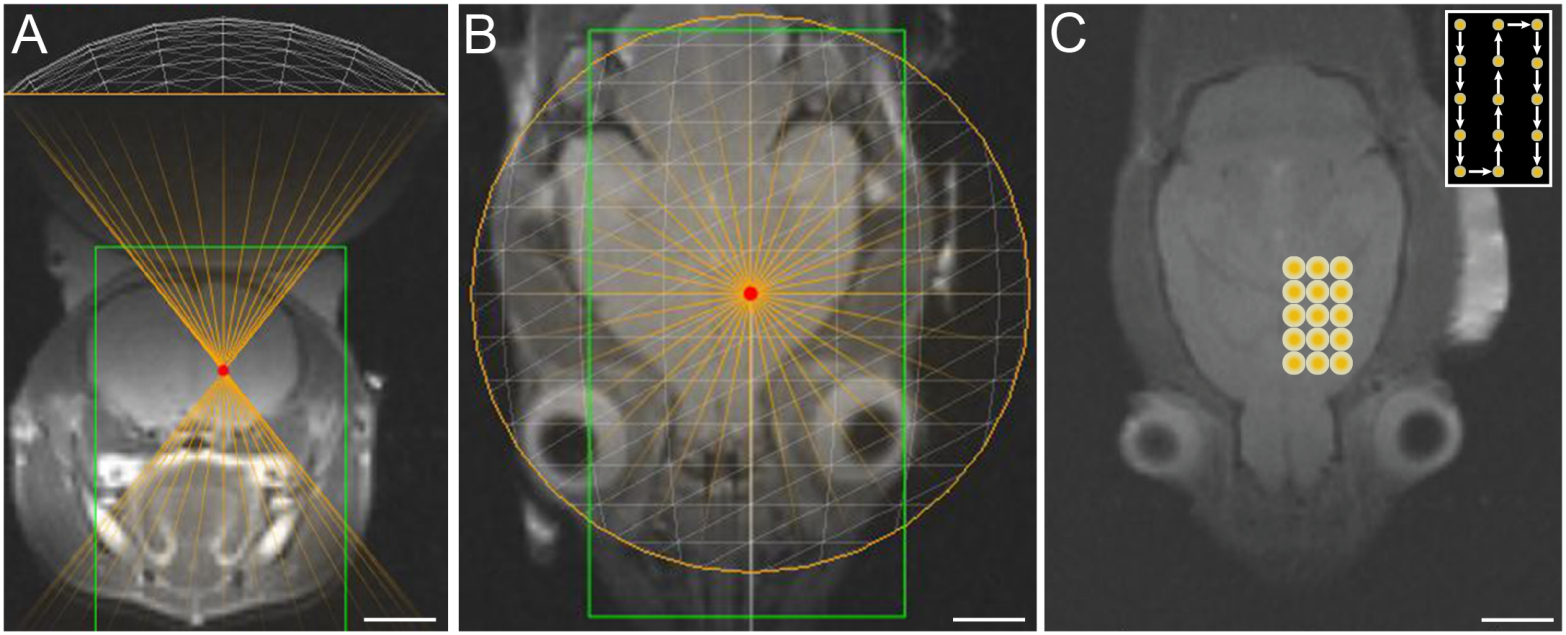
MRgFUS treatment planning. T2-weighted (A) coronal and (B) axial MR images acquired following positioning of the FUS transducer. (C) T2-weighted axial MR image depicting a schematic of the rastering protocol that was used for sonication of the striatum. Each raster point underwent sonication for a total duration of 120 s. Inset depicts a schematic of the order in which each point was treated.

**Fig 4:**
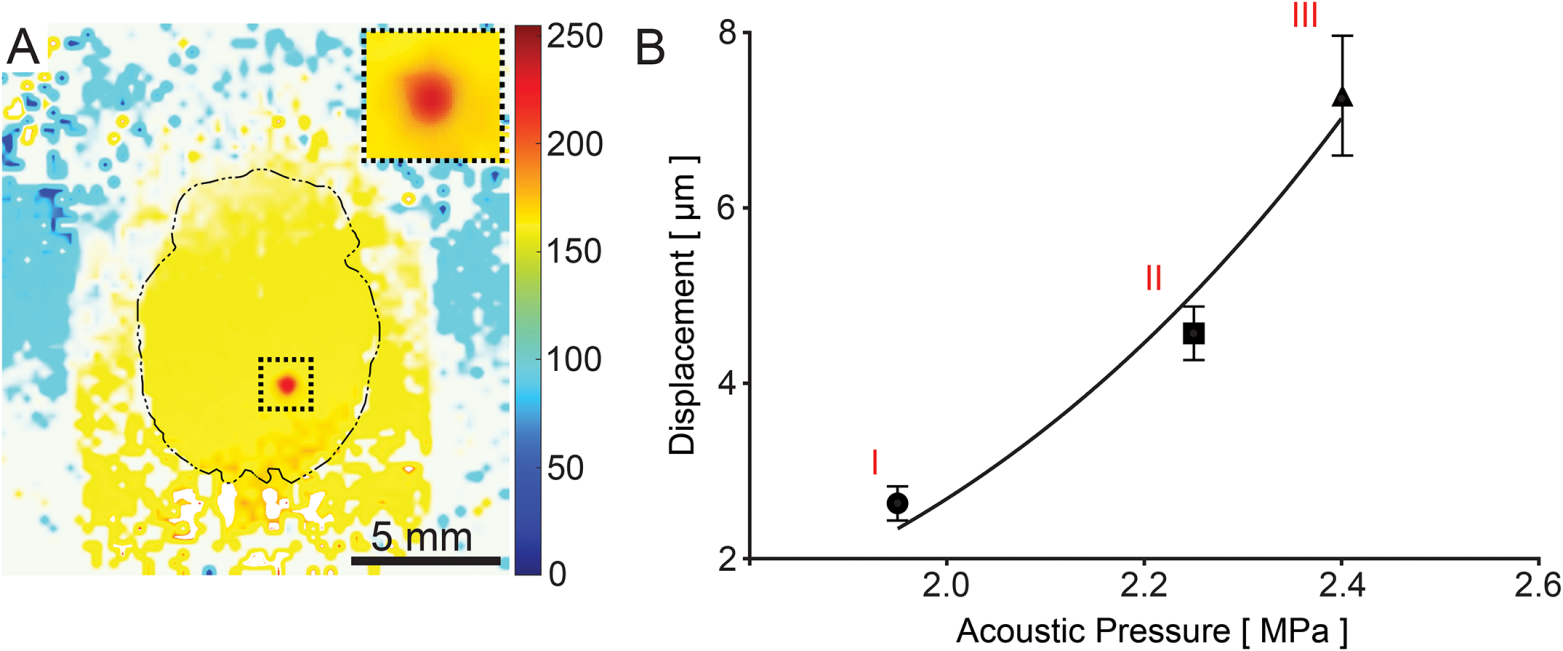
Confirmation of targeting *in vivo* by MR-ARFI imaging. (A) MR-ARFI color map generated by post-processing the MR planes following sonication. Inset depicts sonicated region under higher magnification. (B) Brain tissue displacements generated by MRgFUS pulses and measured by MR-ARFI imaging at acoustic pressures of (I) 1.95 MPa, (II) 2.25 MPa, and (III) 2.4 MPa (n = 5 per acoustic pressure). Error bars represent standard deviation.

A rastering scheme that incorporated 15 discrete and contiguous treatment points was used to treat a volume that measured 7.5 mm × 4.5 mm × 10 mm (*x*, *y*, and *z*, respectively) and was centered on the striatum, maximizing the treatment volume while minimizing the duration of treatment (Fig 3). For verification of targeting accuracy, we used MR-ARFI imaging, a remote-sensing elastography technique that allows for the detection of radiation force-induced displacements in the targeted tissue at a micrometer scale and hence can be used to determine the mechanical properties of tissues [31]. MR-ARFI imaging was used *in vivo* to measure brain tissue displacement following FUS at acoustic pressure levels of 1.95, 2.25, or 2.4 MPa (n = 5 per acoustic pressure). The location of the displacements measured by MR-ARFI imaging corresponded to the targeted region depicted by the graphic user interface (GUI) of the MRgFUS system (Fig 4*A*). Due to the Gaussian distribution of the energy in the focal zone and the viscoelastic properties of the tissue, each sonication produced a gradient of displacement, with the peak displacement located at the center of the beam, and relatively lower levels of displacement at adjacent sites. For the 3 exposures evaluated, we found a mean displacement of 2.63 μm ± 0.19, 4.57 μm ± 0.3, and 7.25 μm ± 0.67, respectively (Fig 4*B*).

### Effect of transcranial MRgFUS pre-treatment on dispersion of locally delivered Evans blue dye and nanoparticles

**Fig 5:**
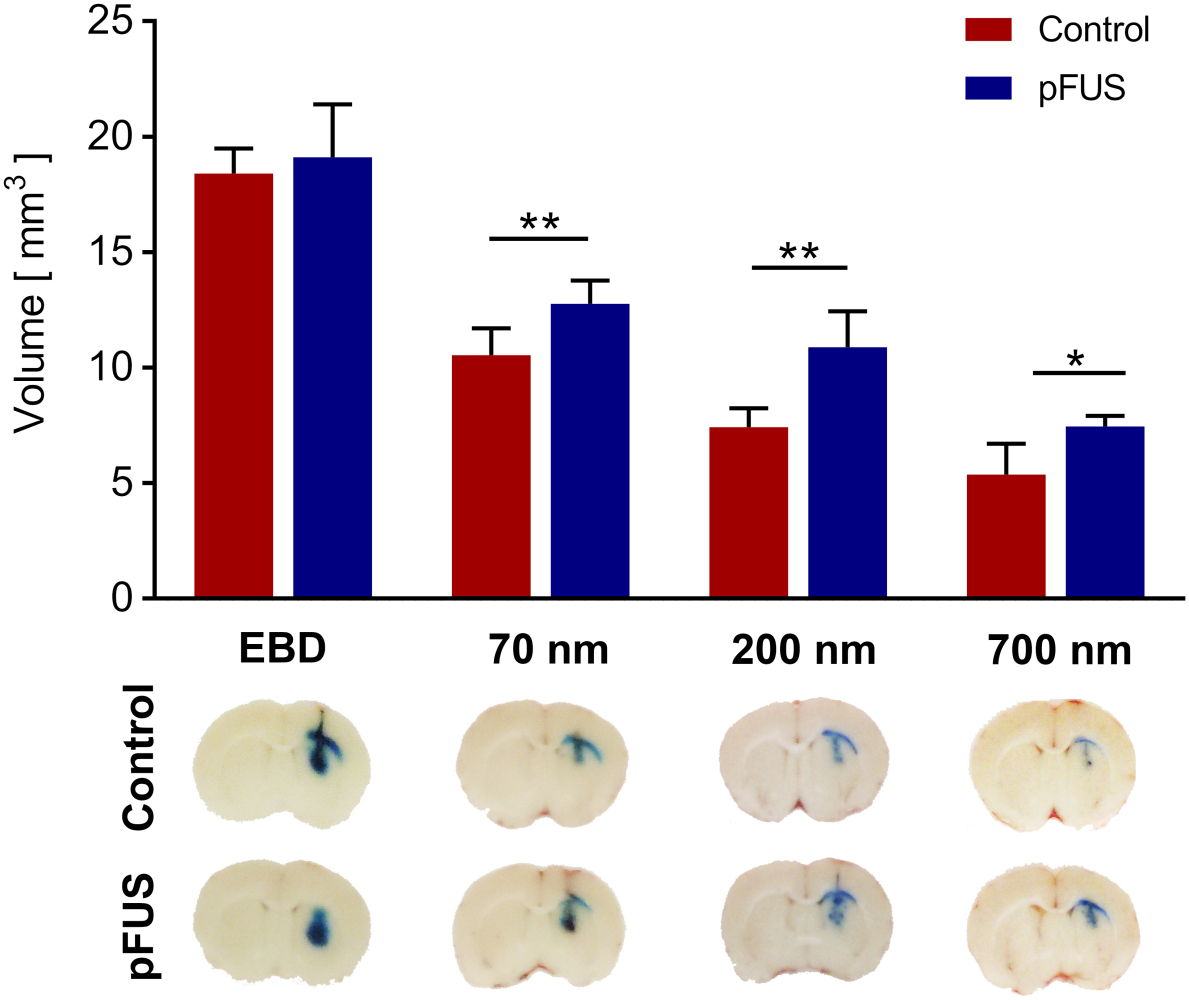
Effect of MRgFUS pre-treatment on the volume of distribution of locally delivered EBD and nanoparticle probes. (Upper) MRgFUS pre-treatment did not have a significant effect on the volume of distribution of EBD, but resulted in statistically significant increases in the volume of distribution of locally delivered 70 nm, 200 nm, and 700 nm blue-dyed, non-adhesive nanoparticles (n = 4–6 treated animals and 4–6 control animals per probe). * p < 0.05, ** p < 0.01. Error bars represent standard deviation. (Lower) Representative images of brain slices obtained 2 hours following the local delivery of EBD or nanoparticles, with or without MRgFUS pre-treatment.

EBD and nanoparticles of varying diameters were locally delivered to the striatum of MRgFUS-treated (n = 4–6) and control (n = 4–6) animals, in order to probe the effects of sonication on the brain interstitium. A MATLAB^™^ (MathWorks, Natick, MA) script was used to measure the volume of distribution of the locally delivered infusate using sequential brain slice images (S1 and S2 Figs). EBD did not exhibit a significantly different volume of distribution in MRgFUS-treated vs. control animals (19.1 ± 2.1 mm^3^ vs. 18.4 ± 0.9 mm^3^, p = 0.64) (Fig 5). However, MRgFUS pre-treatment significantly increased the volume of distribution of 70 nm (12.8 ± 0.9 mm^3^ vs. 10.6 ± 1.0 mm^3^, p = 0.01), 200 nm (10.9 ± 1.4 mm^3^ vs. 7.4 ± 0.7 mm^3^, p = 0.01), and 700 nm (7.5 ± 0.4 mm^3^ vs. 5.4 ± 1.2 mm^3^, p = 0.02) nanoparticles.

### Histological effects

**Fig 6:**
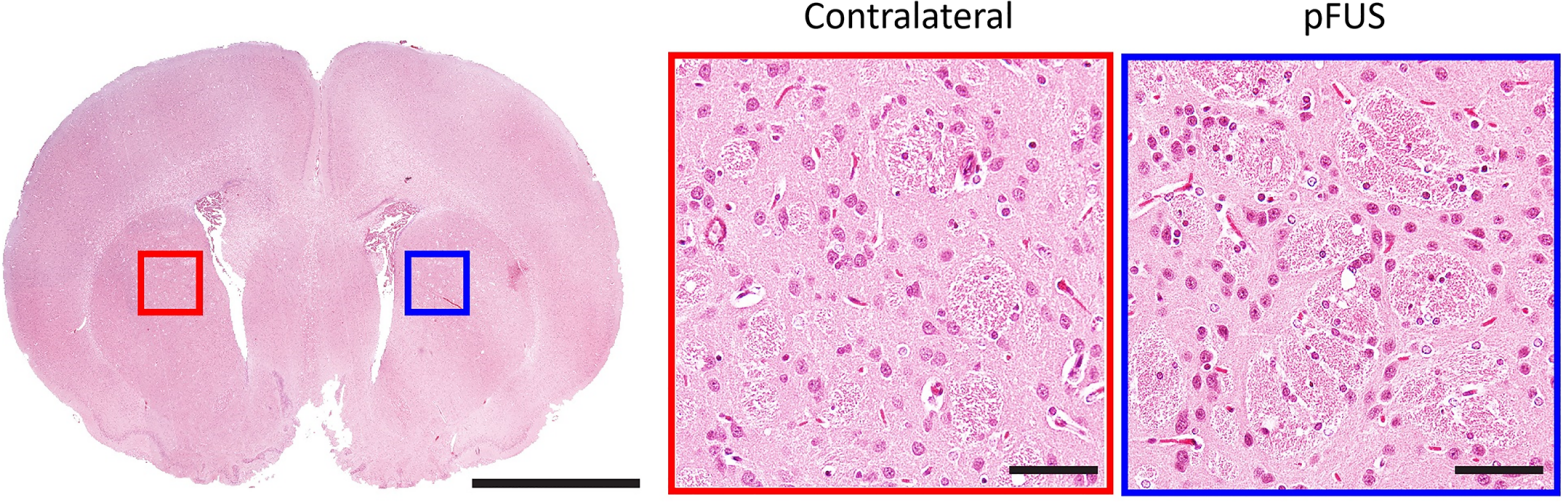
Histological analysis of MRgFUS-treated brains. A representative brain slice obtained 2 hours following MRgFUS treatment of the left striatum indicating a lack of visible differences between the treated (blue) and untreated (red) regions. Left scale bar = 250 μm, center and right scale bars = 25 μm.

In order to determine the effect of sonication on the structural integrity of brain tissue at a histological level, we prepared sections from the brains of animals that were euthanized 2 hours after the completion of the MRgFUS treatment (n = 4), and stained them with hematoxylin and eosin (H&E). These sections were examined by a neuropathologist who was blinded to the status of the treated and untreated hemispheres. Neither overt tissue disruption, pyknotic nuclei, nor red blood cell extravasation was identified in any of the animals (Fig 6).

### Electrophysiological effects

The effect of sonication on the electrophysiological properties of the membrane and synaptic properties of striatal neurons was also investigated. Brain slices from animals euthanized following MRgFUS treatment (n = 5) and from control animals (n = 5) were used for whole-cell recordings from visually identified neurons, in both bridge and voltage clamp (–70 mV) modes.

**Fig 7:**
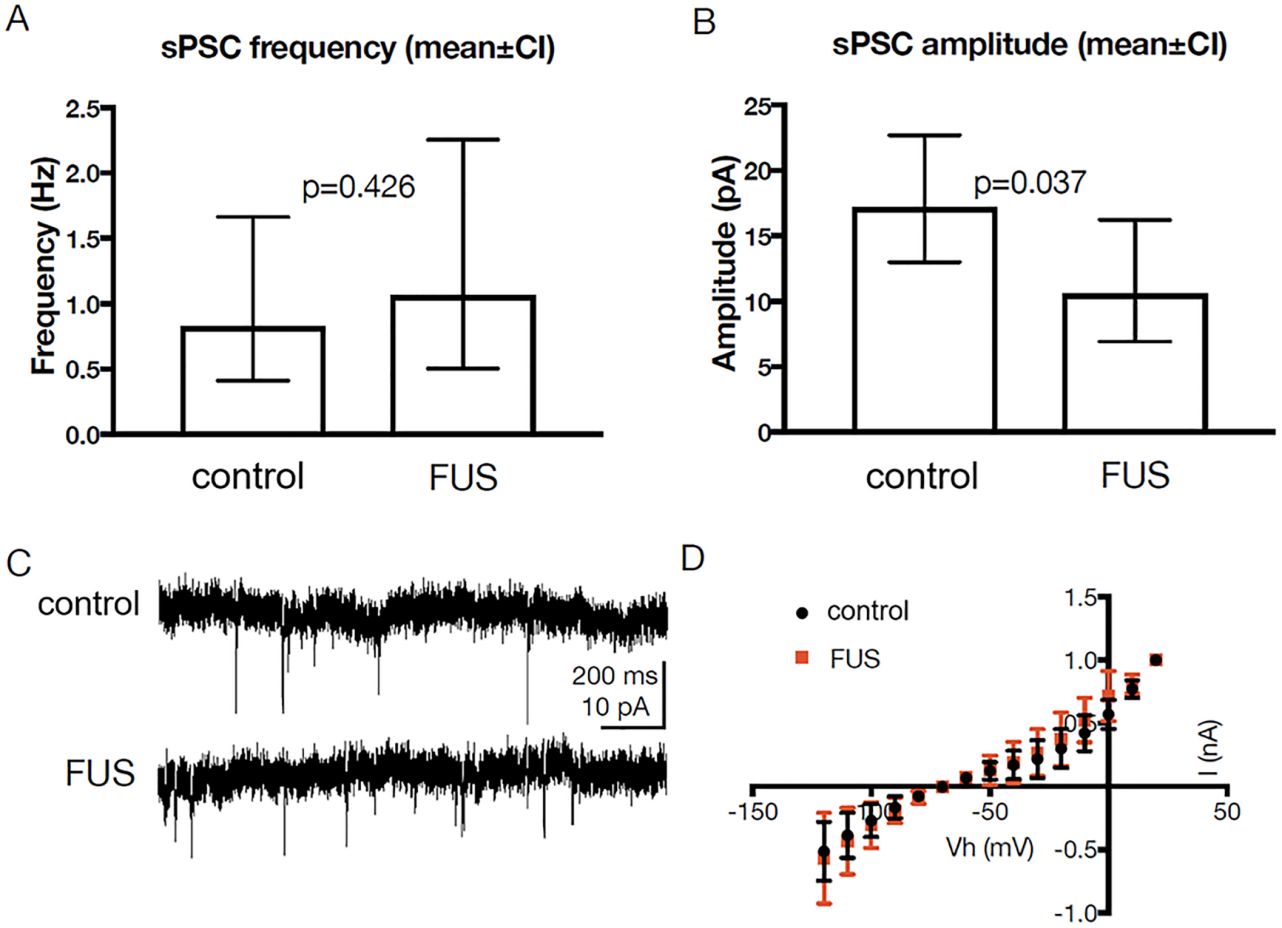
Electrophysiological effects of MRgFUS. (A) MRgFUS did not result in significant differences in the mean frequency of sPSCs recorded from treated animals (n = 13 neurons), compared to those from control animals (n = 10). (B) There was a significant reduction in the mean amplitudes of sPSCs recorded from these two groups. (C) Sample traces of sPSCs recorded from MRgFUS-treated and control animals. (D) Averaged I/V curves recorded from both groups (n = 15 each) indicate that there were no significant differences in whole-cell currents. Error bars represent standard deviation.

To assess synaptic activity we recorded epochs of at least 15 min of spontaneous postsynaptic currents (sPSCs) from neurons of MRgFUS-treated (n = 13) and control (n = 10) rats. There was no significant difference in the mean frequency of these sPSCs (p = 0.43; t = 0.81, df = 21) (Fig 7*A*). In contrast, there was a significant decrease in the mean amplitude of sPSCs recorded from pFUS-treated rats, compared to controls (p = 0.04; t = 2.23, df = 21) (Fig 7*B*). The effect size was large, as determined from Cohen’s d (3.14).

To estimate whole-cell current we applied voltage commands (-120 to 20 mV, 10 mV steps) to the recorded neurons and measured the resulting steady states currents (Fig 7*C* and 7*D*). A two-way analysis of variance (ANOVA) demonstrated no main effect of treatment (F (1, 193) = 0.88, p = 0.35) and no significant interaction (F (14, 193) = 0.55, p = 0.90), suggesting that treatment had no significant effect on whole-cell currents. Estimation of Rin from these traces revealed no significant differences between the groups (p = 0.56; t = 0.59, df = 22).

We used recordings in bridge mode to test for differences in resting membrane potential and in rheobase (the minimal current resulting in action potential generation). We found no significant difference in either resting membrane potential (p = 0.66, t = 0.45, df = 21) or rheobase (p = 0.1, t = 1.73, df = 18).

## Discussion

The recent FDA approval of MRgFUS for the thermal lesioning of dysfunctional neural circuits deep within the brain has introduced a new clinical tool for the modulation of brain tissues and the treatment of neurological diseases. Ongoing clinical trials in patients with brain tumors and Alzheimer’s disease using MB-based BBB disruption are seeking to advance the non-thermal applications of MRgFUS using pulsed exposures. In this study, we utilized a small animal MRgFUS system to study transcranial FUS without MB augmentation, thereby directing the effects toward the brain interstitium. Extrapolating from exposures that had previously been shown to expand the ECS in a non-destructive manner within muscle [23] and solid tumor xenografts [26], we observed similar results *in vivo* to those found in our *ex vivo* study [27], including increased dispersion of nanoparticle probes without evidence of injury to the treated tissue. Computer simulations were performed to optimize the treatment scheme, and MR-ARFI imaging enabled confirmation of the intracranial targets, highlighting the potential value of this new method for accurate treatment execution of non-thermal ultrasound regimens. Taken together, our results suggest that MRgFUS can be safely and accurately applied through the intact skull in pulsed, non-thermal modes. The resulting expansion of the brain interstitium, as evidenced by increased nanoparticle dispersion [27, 34, 38], has important implications for drug and particulate delivery in the brain.

The findings of this study support the observations from our previous work, where enlargement of the ECS was observed in skeletal muscle [23], solid tumors [26], and brain tissue *ex vivo* [27], as well as similar work examining expansion of the fibrin matrix of acute blood clots [39]. These effects were subsequently shown to enhance the spread of fluorescently labeled agents including nanoparticles [21, 23, 40], lectins [23], and monoclonal antibodies [41] within the respective tissues. When substituting the fluorescent probes for therapeutic versions, growth inhibition was facilitated in solid tumor xenografts [26, 41, 42] and thrombolysis was enhanced in acute [43, 44] and chronic [45] blood clots. Similarly, in the current study we found increased dispersion of large nanoparticle probes following transcranial MRgFUS, significantly larger than the reported size limit for unhindered movement of particulates within the mammalian brain [38, 46]. While others have investigated the effect of transcranial FUS on the spread of locally delivered tracers, in those cases FUS was applied concurrently with the tracers [18, 35, 47, 48] or in the presence of intravascular MBs [49]. As a result, ultrasound mechanisms related to blood vessel disruption or convective forces such as acoustic streaming likely contributed to the observations of improved dispersion [24]. This study demonstrates the potential for transcranial MRgFUS to specifically modulate the microstructure of the brain without causing significant changes in neuronal function.

In the current study, MRgFUS pre-treatment significantly increased the volume of distribution of 70 nm, 200 nm, and 700 nm nanoparticles, but not that of EBD. One explanation is that with a molecular weight of 961 Da, EBD encounters minimal steric hindrance, thereby diffusing efficiently through the brain ECS, and is not significantly affected by the size of the ECS. Similarly, in our prior study in skeletal muscle, fluorescently labeled albumin was co-injected with 100 nm nanoparticles [23]. Albumin, a small molecule, had a larger area of distribution than the nanoparticles within the tissue, as did the EBD in the current study. However, FUS did not affect the distribution of the albumin due to its small size, whereas the distribution of the 100 nm nanoparticles was significantly increased. In both cases, the albumin and EBD distributions were substantially greater (~ 2-fold) than those of nanoparticles used in the respective studies.

The MRgFUS exposures used in the current study were not associated with evidence of histological damage and minimal electrophysiological alterations. Although we have previously validated the safety of FUS outside the brain [23, 26, 40–43], prior studies in the brain, particularly those that have investigated the use of FUS with MBs, have tended to investigate only the structural changes that are induced by sonication [50–52]. Such studies have used histological analysis to assess for capillary damage, micro-hemorrhages, macrophage infiltration, cyst cavity formation, or scar formation. However, FUS may have more subtle effects on neuronal tissue, resulting in alterations that are not histologically evident. McDannold et al. [53] were the first to explore electrophysiological function as a means of assessing the safety of transcranial FUS. MB-enhanced FUS was used to non-thermally ablate skull base targets adjacent to the optic chiasm or tract, and visual evoked potentials were used to confirm that the neural function of the optic apparatus remained unaffected. In the current study, we expanded on this concept and performed an in-depth analysis of the effect of MRgFUS on the electrophysiological properties of the cell membrane, as well as the synaptic properties of striatal neurons. Although a decrease in the mean amplitude of sPSCs was observed, there was no effect on the mean frequency of the sPSCs. The decrease in the amplitude of spontaneous synaptic currents, but not in their frequency, is consistent with postsynaptic changes intrinsic to the recorded neurons [54]. Although we did not detect significant changes in input resistance after MRgFUS treatment, we cannot exclude the possibility that reductions in input resistance at distal dendrites, that cannot be reliably detected with somatic recordings [55], contributed to the decrease in the amplitude of synaptic currents. These amplitude changes may also reflect changes in properties of transmitter receptors that were not directly assayed in the present study. However, MRgFUS had no effect on whole-cell currents, resting membrane potential, or rheobase. Therefore, our data indicate that MRgFUS has minimal effects on the microstructure and electrophysiological function of the striatum, despite generating effects that lead to an increase in the volume of distribution of locally delivered nanoparticles. Additional studies of this application may further improve its optimization and safety.

The proposed mechanism by which FUS exposures alter the ECS is through the generation of unidirectional radiation force-induced displacements in the direction of the propagating wave. These occur due to a transfer of momentum from the ultrasound wave to the tissue being treated [56, 57]. Each FUS pulse can generate displacements as large as 200 μm, peaking at the center of the focal zone, where the energy is concentrated [23]. On the margins of the focal zone, at the interface between tissue that is and is not being actively displaced, shear stress is maximized, resulting in comparatively large shear strain. This strain, in turn, affects the relatively weak cell-to-cell interfaces within the tissue, as well as the extracellular matrix, resulting in enlargement of the ECS [23]. In the current study, displacements of 4.57 μm were found to occur for each individual pulse, where a total of 1200 pulses were given at each treatment location.

Real-time monitoring of the *in vivo* effects of FUS is a critical safety feature in the eventual clinical translation of new therapeutic regimens [58]. This is evidenced by the critical role of real-time MR thermometry in the clinical MRgFUS systems currently used in the treatment of Essential Tremor, where relatively high rates of energy deposition are applied and the mechanism of action of the FUS exposures is thermal ablation. MR thermometry is not only used to validate that a sufficient thermal does has been applied to a target, but also that adjacent and intervening tissues have not been exposed to undesired heating effects [28, 59]. Acoustic emissions monitoring using hydrophones is another valuable tool employed in the clinical system to detect acoustic cavitation [60, 61], which can lead to severe and uncontrolled tissue injury.

The MR-ARFI monitoring method investigated in this study is a potential new tool for real-time, *in vivo* monitoring of the radiation force-induced displacements produced by non-thermal transcranial FUS regimens. Prior studies have shown that the degree of tissue displacement and the resulting shear strain are directly proportional to the intensity of the FUS exposure and the mechanical properties, primarily the elastic modulus, of the treated tissue [56, 57]. Extrapolating from our earlier studies in muscle [23] and solid tumors [26], we employed an acoustic pressure for the FUS exposures where comparable displacements would be generated in the brain parenchyma. We then used MR-ARFI imaging measurements to validate the location and degree of displacement. Prior studies have explored MR-ARFI imaging as a means of providing real-time feedback for adaptive focusing and correction of phase aberrations caused by the skull during transcranial sonication [62, 63]. In that setting, MR-ARFI is used as an alternative to MR thermometry. In the setting of pulsed FUS, however, temperature elevations are minimal. Additionally, in the absence of FUS-MB induced disruption of the BBB, intravenous contrast enhancement cannot be used as a means of validating the localization of the sonicated target. Instead, MR-ARFI imaging may be used to directly measure the radiation force-induced displacements produced by FUS *in vivo*, where relatively short pulses are employed. In this study, the relationship between the applied acoustic pressure and the resulting displacements was observed to be exponential, similar to previous reports [31]. Furthermore, the specific acoustic pressure employed for the MR-ARFI imaging measurements was below that used for the treatments, indicating that this procedure can be safely employed for its stated purpose.

This study has several limitations that will be addressed in future work. Although we investigated the effect of MRgFUS on multiple domains of central nervous system (CNS) structure and function, the downstream molecular effects of FUS have yet to be determined. In particular, disruption of the BBB via FUS in conjunction with MBs has recently been shown to induce a sterile inflammatory response [64]. Nevertheless, it is unclear whether a similar response occurs following FUS in the absence of MBs. Furthermore, we studied a selected set of MRgFUS parameters, but other FUS exposures should be explored in an effort to further optimize the effects on the brain ECS while minimizing any negative structural and functional consequences. Additionally, while we explored the immediate effects of transcranial MRgFUS, future studies should examine long-term structural and neurophysiological effects, including their potential for reversibility over time. Lastly, the process of inserting a syringe for *in vivo* infusions inherently disrupts the surrounding tissue, resulting in some flow of infusate along the insertion tract and along white matter tracts. Although this phenomenon has been described in other studies [17, 35], future work may examine the effect of transcranial MRgFUS on the distribution of nanoparticles that are delivered to intact tissue.

In summary, this study demonstrates that transcranial MRgFUS can safely and effectively enhance the interstitial dispersion of large polymeric nanoparticles in the living brain, further reinforcing the tissue-modulating potential of this technology. Future studies characterizing the therapeutic window of the induced effects, as well as the molecular and physiologic changes induced by this pFUS regimen in the brain, will provide additional information related to the full potential of MRgFUS in the treatment of neurological diseases.

## Supporting information

S1 Fig Automated segmentation of locally delivered EBD and nanoparticles using a MATLAB algorithm. (A-C) Representative images of brain slices obtained from an animal 2 hours following the infusion of EBD. (D-F) Following an automated normalization process to account for differences in ambient light intensity, correction factors were applied to each image. (G-I) The RGB images were converted to grayscale, and automated Otsu thresholding was used to identify the optimal threshold for each image, enabling the calculation of the total volume of distribution.
(TIF)

S2 Fig 3D volumetric reconstructions. Representative, three-dimensional nanoparticle (200 nm) distribution volumes from control and MRgFUS-treated brains. The distributions were reconstructed from individual sections captured as digital images and used for quantitative comparisons between control and MRgFUS-treated groups. The arrows indicate the location of the injections.
(TIF)

S1 Checklist ARRIVE guidelines checklist. (PDF)
