# Supplementary figures and images for: MR-guided transcranial focused ultrasound safely enhances interstitial dispersion of large polymeric nanoparticles in the living brain
Source: PLoS One. 2018 Feb 7;13(2):e0192240. doi: 10.1371/journal.pone.0192240 (PMC5802894; doi:10.1371/journal.pone.0192240)

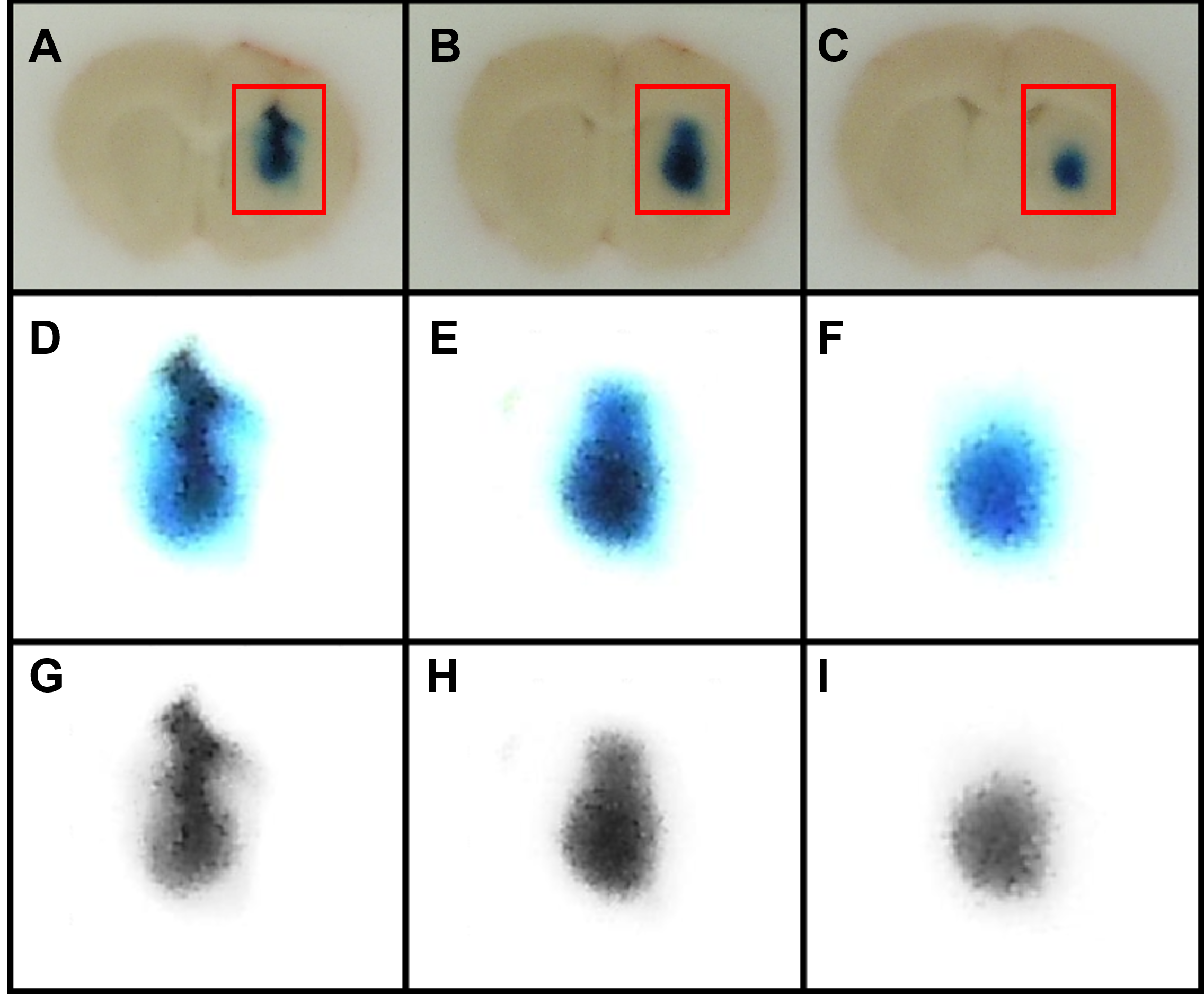

Supplement: S1 Fig — (A-C) Representative images of brain slices obtained from an animal 2 hours following the infusion of EBD. (D-F) Following an automated normalization process to account for differences in ambient light intensity, correction factors were applied to each image. (G-I) The RGB images were converted to grayscale, and automated Otsu thresholding was used to identify the optimal threshold for each image, enabling the calculation of the total volume of distribution. (TIF) [file pone.0192240.s001.tif]

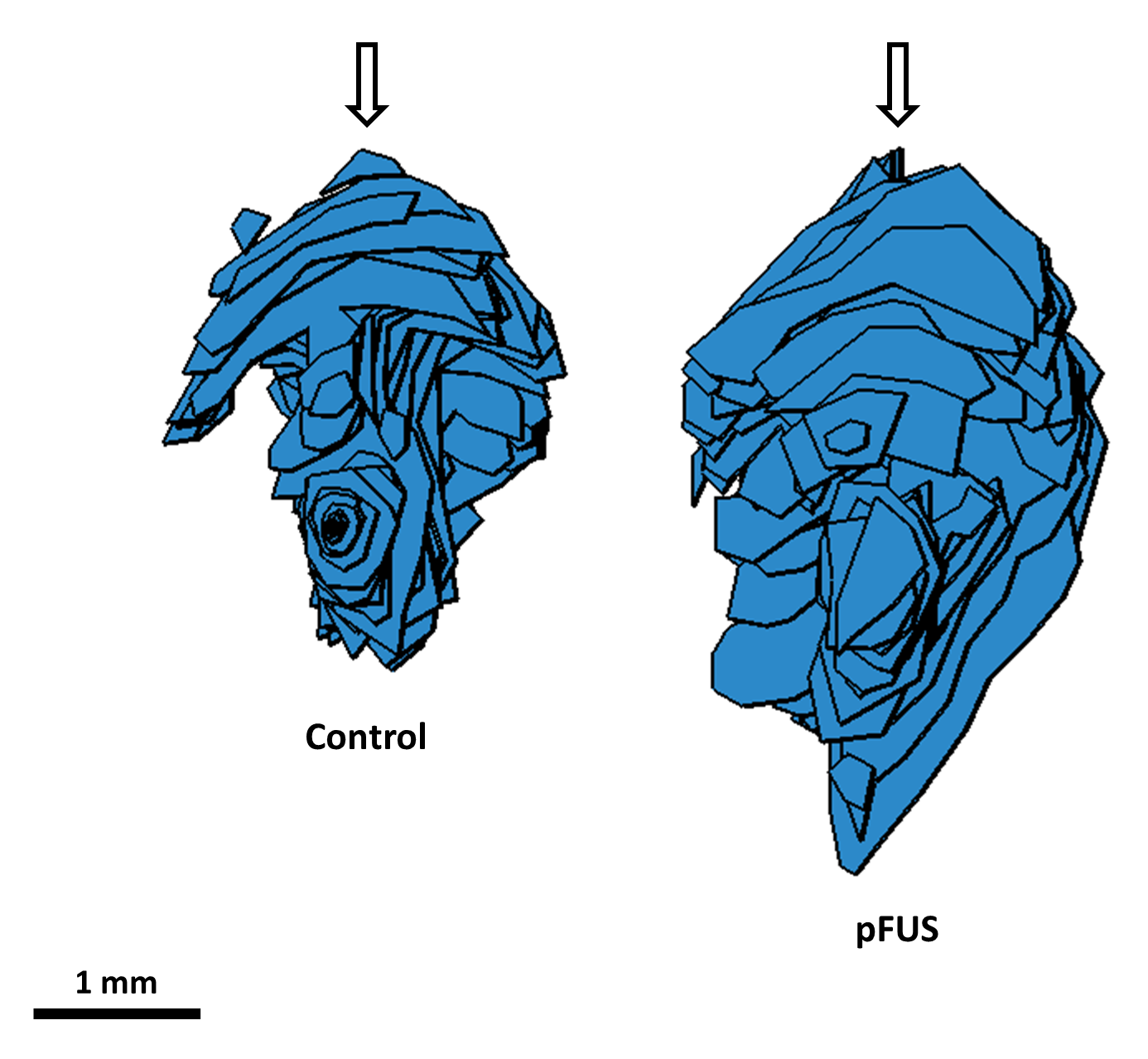

Supplement: S2 Fig — Representative, three-dimensional nanoparticle (200 nm) distribution volumes from control and MRgFUS-treated brains. The distributions were reconstructed from individual sections captured as digital images and used for quantitative comparisons between control and MRgFUS-treated groups. The arrows indicate the location of the injections. (TIF) [file pone.0192240.s002.tif]
